# Obituary—Dr. S. L. Hamlin

**Published:** 1866-07

**Authors:** 


### OBITUARY.

Died in the city of New York May 14th, 1866, Dr. S. L. Hamlen formerly a Dentist in this city.

Many years ago when Dr. Hamlen first came to this city, (Cincinnati,) he united himself in business with Dr. M. Rogers whom he suceeded in business some three years later at No. 3 West 4th Street, where he continued to practice until four years ago, when from failing health he was forced to retire from the Profession.

By persevering energy, close attention to business and a love for his profession, he built up a large and lucrative practice.

The last four years of active business in the open air recruited his failing energies, and seemed to promise him a new lease on life.

But he soon tired of his new business, and sought again his Profession.

Only a few weeks before his death he opened a Dental Office at No. 22 Bond Street, New York, with good prospects of success before him.

Though apparently fully restored to his former health an insidious disease was still lurking in his system, which at last ended his life.

